# Fuzheng Huayu Recipe Prevented and Treated CCl4-Induced Mice Liver Fibrosis through Regulating Polarization and Chemotaxis of Intrahepatic Macrophages via CCL2 and CX3CL1

**DOI:** 10.1155/2020/8591892

**Published:** 2020-11-25

**Authors:** Man Zhang, Hong-liang Liu, Kai Huang, Yuan Peng, Yan-yan Tao, Chang-qing Zhao, Xu-dong Hu, Cheng-hai Liu

**Affiliations:** ^1^Institute of Liver Diseases, Shuguang Hospital Affiliated to Shanghai University of Traditional Chinese Medicine, 528 Zhangheng Road, Pudong New Area, Shanghai 201203, China; ^2^Department of Biology, School of Basic Medical Sciences, Shanghai University of Traditional Chinese Medicine, 1200 Cailun Road, Pudong New Area, Shanghai 201203, China; ^3^Shanghai Key Laboratory of Traditional Chinese Clinical Medicine, 528 Zhangheng Road, Pudong New Area, Shanghai 201203, China

## Abstract

**Background:**

Fuzheng Huayu recipe (FZHY) is an original Chinese patent medicine which was developed and marketed by our institute. It could markedly improve liver tissue inflammation and ameliorate hepatic fibrosis in the clinical study. The intrahepatic macrophages recruitment and polarization play an important role in the progress of liver inflammation and fibrosis. Whether FZHY exerted its antiliver fibrosis effects through regulating intrahepatic macrophages phenotypic ratios is still unknown. This study aims to explore the antifibrosis mechanism of FZHY on regulating the recruitment and polarization of intrahepatic macrophages.

**Methods:**

C57/B6 mice were used for the establishment of the CCl4-induced mice liver fibrosis model. Liver inflammation and fibrosis were evaluated by HE and Sirius red staining, hydroxyproline assays, and biochemical tests. The levels of chemokines and inflammatory cytokines in liver tissue were measured by RNA-Seq transcriptome analysis, western blot assay, RT-qPCR, and immunofluorescence assay. The macrophages recruitment and phenotypic polarization were observed by flow cytometry.

**Results:**

FZHY significantly improved liver inflammation and reduced liver fibrosis degree. TNF signaling pathway, involved in macrophages recruitment and phenotypic polarization, was discovered by RNA-Seq transcriptome analysis. In TNF signaling pathway, CCL2 expression was significantly decreased and CX3CL1 expression was significantly upregulated by FZHY in liver tissue and primary intrahepatic macrophages. The ratio of proinflammatory hepatic resident macrophage-Kupffer cells (F4/80^+^CD11b^−^CD86^+^) was downregulated by FZHY, while the proportion of anti-inflammatory Kupffer cells (F4/80^+^CD11b^−^CD206^+^) was upregulated. Meanwhile, the ratio of proinflammatory Ly6C^high^ macrophages (F4/80^+^CD11b^+^Ly6C^high^) which were recruited from blood circulation by CCL2 was reduced by FZHY, while the ratio of restorative Ly6C^low^ macrophages (F4/80^+^CD11b^+^Ly6C^low^) which were recruited from blood circulation or induced from Ly6C^high^ macrophages polarization by CX3CL1 was significantly increased.

**Conclusions:**

FZHY could regulate the recruitment and polarization of intrahepatic macrophages via CCL2 and CX3CL1, so as to play its anti-inflammation and antifibrosis pharmacological effects in the liver.

## 1. Introduction

Liver fibrosis is the common pathological basis of the development of many chronic severe liver diseases and is also the early and inevitable stage of liver cirrhosis. It is a pathological process caused by continuous injury-repair response. Hepatocytes are induced to apoptosis and necrosis by a variety of chronic liver diseases, such as viral hepatitis, metabolic liver disease, fatty liver disease, and autoimmune liver disease, and then intrahepatic macrophages are activated and recruited. The activated and recruited intrahepatic macrophages produce a great number of inflammatory cytokines, such as tumor necrosis factor (TNF)-*α* and interleukin (IL)-6, to aggravate the damage of hepatocytes and stimulate the activation of hepatic stellate cells (HSCs). Activated HSCs are transformed into proliferative myofibroblasts which will lead to excessive deposition of extracellular matrix and the formation of liver fibrosis by secreting large amounts of collagen [1–4].

In the process of chronic liver diseases, the IFC axis (inflammation ⟶ fibrosis ⟶ cancer) has attracted more and more attention [5]. Chronic liver inflammation is the prerequisite for inducing liver fibrosis [6]. Intrahepatic macrophages are the most important immune- and inflammation-related cells in the liver inflammation. They are the main source cells of inflammatory cytokines and chemokines and play an important role in the process of liver fibrogenesis [7]. According to their origins, intrahepatic macrophages are divided into two groups: hepatic resident macrophages, Kupffer cells (KCs), which reside in the liver, and peripheral mononuclear-derived macrophages which are recruited from peripheral blood. Several studies with experimental mouse models have showed that the recruited monocytes have the crucial role on the progression of liver inflammation and fibrosis [8, 9]. Peripheral blood monocytes can constantly replenish to generate Kupffer cells, especially in acute or chronic liver injury [10]. Chemokines play an important role on monocytes recruitment. Chemokine monocyte chemotactic protein 1 (CCL2), which is mainly released by proinflammatory macrophages in the liver, recruits bone marrow-derived proinflammatory Ly6C^high^ monocytes from the blood to the liver through recognizing receptor CCR2 on proinflammatory Ly6C^high^ monocytes cell membrane. Proinflammatory Ly6C^high^ monocytes will develop into proinflammatory Ly6C^high^ macrophages. Then, proinflammatory Ly6C^high^ macrophages together with proinflammatory Kupffer macrophages further aggravate the inflammatory injury of the liver by secreting proinflammatory factors. Fractalkine (CX3CL1), which is released by several cells such as hepatic endothelial cells and monocytes [11] in the liver, recruits bone marrow-derived restorative Ly6C^low^ monocytes from the blood to the liver through recognizing receptor CX3CR1 on restorative Ly6C^low^ monocyte cell membrane. Restorative Ly6C^low^ monocytes will develop into restorative Ly6C^low^ macrophages. CX3CL1 also can switch proinflammatory Ly6C^high^ macrophages to restorative Ly6C^low^ macrophages. Restorative Ly6C^low^ macrophages show anti-inflammatory and tissue-protective features and exert their role on anti-inflammation and the promotion of liver tissue injury repair and finally inhibit the progress of liver fibrosis [12, 13].

Fuzheng Huayu recipe (FZHY), developed by our institute (Institute of Liver Diseases), is an effective original Chinese patent medicine for liver fibrosis. It is composed of Radix Salvia Miltiorrhizae, Semen Persicae, Cordyceps Sinensis Mycelia, Pollen Pini, Fructus Schisandrae, and *Gynostemma Pentaphyllum*. The clinical research results of FZHY against chronic hepatitis B-related liver fibrosis have showed that FZHY could significantly improve the inflammation of liver tissue while ameliorating hepatic fibrosis without adverse reactions [14]. Experiment results also showed that FZHY could inhibit the production of TGF-*β* and PDGF in primary Kupffer cells (KCs) from CCl4-injured rats [15]. Furthermore, FZHY could inhibit the activation of hepatic stellate cells by regulating the paracrine of KCs [16]. The above results suggest that the antiliver fibrosis effect of FZHY is related to its function of regulating macrophages polarization. But, the mechanism underlying is still unclear. This study was trying to elucidate whether FZHY exerted its anti-inflammation and antifibrosis effects through regulating intrahepatic macrophage polarization and recruitment.

## 2. Materials and Methods

### 2.1. Reagents

Carbon tetrachloride (CCl4, Cat No: 10006418) and olive oil (cat no: 69018028) were purchased from Sinopharm Co., Ltd, China. Hydroxyproline (Hyp), alanine transaminase (ALT) (cat no: C009), and aspartate transaminase (AST) (cat no: C010) kits were purchased from Nanjing-Jiancheng biological engineering research institute, China. An RNA simple Total RNA Kit was purchased from Tiangen Biotech, China. A ReverTra Ace qPCR RT Kit was purchased from Toyobo, Osaka, and Japan. RIPA buffer and BCA protein quantification kits were purchased from Beyotime Biotechnology, China. Nitrocellulose (NC) membrane (cat no: RPN303C) was purchased from Hybond, USA. Pronase E, collagenase IV, DNase I, and Histodenz were purchased from Sigma, USA. Anti-F4/80 immunohistochemical antibody (cat no: ab16911) was purchased from Abcam, UK. Anti-CCL2 immunohistochemical and western blot antibody (cat no: 25542-1-AP) was purchased from Proteintech, USA. Anti-CX3CL1 western blot antibody (cat no: 14-7986-81) was purchased from eBioscience, USA. Anti-GAPDH western blot antibody (cat no: 60004-I-Ig) was purchased from Proteintech, USA. Fluorescent second antibodies, goat anti-rabbit IgG H&L (FITC) (cat no: ab6717), and donkey anti-rat IgG H&L (Alexa Fluor 647) (cat no: ab150155) were purchased from Abcam, UK. DAPI (cat no: ab228549) was purchased from Abcam, UK. Flow cytometric antibodies including anti-CD45 (cat no: 557659), anti-CD11b (cat no: 564454), anti-F4/80 (cat no: 565613), anti-Ly6C (cat no: 560525), and anti-CD86 (cat no: 558703) were purchased from BD Pharmingen, USA. Anti-CD206 (cat no: 141720) was purchased from BioLegend, USA.

One dose of Fuzheng Huayu recipe (FZHY) is composed of Radix Salvia Miltiorrhizae (root of Salvia miltiorrhiza Bunge) (4 g), Semen Persicae (Prunus persica (L.) Batsch) (2 g), Cordyceps Sinensis mycelia (8 g), Pollen Pini (pollen of Pinus thunbergii Parl) (2 g), Fructus Schisandrae Chinensis (fruit of Schisandra chinensis (Turcz.) Baill.) (2 g), *Gynostemma pentaphyllum* (Thunb.) Makino (6 g) (SFDA approval number: Z2005050546 and batch number: 180206). The preparation of FZHY extracts was prepared and provided by Shanghai Huanghai Pharmaceutical Co. Ltd. (Shanghai, China) with a quality inspection report, including the chromatographic profile and the contents of adenosine, danshensu, and salvianolic acid B (2.26 mg/g, 8.3 mg/g, and 13.24 mg/g, respectively).

### 2.2. Animal Experiment

Six-week-old C57/B6 male mice (22–25 g) were purchased from Shanghai Lingchang Biotechnology co. Ltd (Shanghai, China). The mice were housed in a specific pathogen-free (SPF) grade and temperature-controlled room (22 ± 2°C) subjected to a 12 h light/dark cycle, with free access to water and food.

All mice were randomly separated into three groups: control group (normal group, *n* = 16), CCl4-induced liver fibrosis group (CCl4 group, *n* = 16), and FZHY-administered group (FZHY group, *n* = 16). Mice in the CCl4 group and FZHY group were administrated with 15% CCl4-injection intraperitoneally (dissolve CCl4 in olive oil, 2 ml/kg) thrice weekly for 6 weeks. At the same time, mice in the FZHY group were orally given FZHY (5.6 g/kg) daily for 6 weeks. Mice in the normal group were administrated with olive oil intraperitoneally and physiological saline orally. At the end of experimental period, mice were anesthetized by inhaling 3% isoflurane. Some of mice were drawn blood quickly through the inferior vena cava, and then their livers were taken and preserved for further testing, followed by cervical dislocation for confirmation of euthanasia. The others were punctured by the portal vein and their livers were perfused, then their intrahepatic macrophages were isolated, and the phenotypic typing of intrahepatic macrophages was observed by flow cytometry.

### 2.3. Measurement of Serum ALT and AST Levels

Serum was obtained by centrifugation after blood collection from the inferior vena cava of mice. According to the operation instructions of ALT and AST test kits, the serum was mixed with the reaction solution containing alanine and *α*-ketoglutarate (for ALT test) and the reaction solution containing *α*-ketoglutarate and aspartic acid (for AST test) for 30 min, respectively. Then, 2,4-dinitrophenylhydrazine (DNPH) hydrochloric acid solution was added to terminate the reaction and produce pyruvate phenylhydrazone. Finally, sodium hydroxide was added to develop the color. The OD value at 505 nm wavelength was read. The contents of ALT and AST in serum were calculated according to the ALT and AST standard curve.

### 2.4. Measurement of Liver Hydroxyproline (Hyp) Level

The liver tissue was homogenized and hydrolyzed in 50% hydrochloric acid at 110°C for 24 h. Then, the sample homogenate was filtered and roasted at 40°C for 48 h to obtain precipitation. Next, the sample precipitation was dissolved in 50% isopropanol. After that, chloramine-T working solution was added and mixed well. Then, the mixture was placed at room temperature for 10 min, and ER working solution was added and bathed in 50°C water for 90 min. Finally, the OD value at 558 nm was read. The content of hydroxyproline (Hyp) in the liver was calculated according to the Hyp standard curve.

### 2.5. Histopathological Examination

Liver tissues were fixed in 4% paraformaldehyde, followed by dehydration and embedding in paraffin, and finally 4 *μ*m thick slices were stained with H&E and Sirius red for histopathological assessing [17].

### 2.6. RNA-Seq

Liver tissue was collected from the control group, CCl4-induced group, and FZHY-treated group. Total RNA was extracted using RNeasy Mini Kit (Cat#74106, Qiagen) following the manufacturer's instructions and checked for a RIN number to inspect RNA integrity by an Agilent Bioanalyzer 2100 (Agilent technologies, Santa Clara, CA, US). Qualified total RNA was further purified by RNA Clean XP Kit (Cat A63987, Beckman Coulter, Inc. Kraemer Boulevard Brea, CA, USA) and RNase-Free DNase Set (Cat#79254, QIAGEN, Gm BH, Germany). Following purification, the mRNA was isolated and fragmented. The cleaved RNA fragments were copied into first-strand complementary DNA (cDNA) using reverse transcriptase and random primers. This was followed by second-strand cDNA synthesis using DNA polymerase I and RNase H. Then, these cDNA fragments were run through an end-repair process, the addition of a single “A” base, and then ligation of the adapters. The products are then purified and enriched with PCR to create the final cDNA library. Purified libraries were quantified by a Qubit 2.0 Fluorometer (Life Technologies) and validated by an Agilent 2100 bioanalyzer (Agilent Technologies) to confirm the insert size and calculate the mole concentration. The cluster was generated by cBot with the library diluted to 10 pM and then sequenced on the Illumina HiSeq X ten (Illumina). The library construction and sequencing were performed at Shanghai Biotechnology Corporation, Shanghai, China.

### 2.7. Data Processing of Transcription Group Sequencing

The raw reads contain unqualified reads such as reads of the low-quality end, the reads including primers. To obtain clean reads for data analysis, these unqualified reads could be filtered by Seqtk screening.

### 2.8. Screening of Differential Expression Genes

The reads are not only proportional to the gene expression level but also related to the length of the gene itself and the amount of data sequenced. In order to obtain comparable data on the gene expression levels of different genes and different samples, the reads were converted into FPKM (fragments per kilobase of exon model per million mapped reads), to standardize the gene expression by three steps which were Stringtie count, TMM (trimmed mean of M values) normalization, and PERL script calculation. Using edgeR to perform differential gene analysis between samples, the *p* value was obtained and the multihypothesis test was performed. The threshold of *p* value was determined by controlling the FDR (false discovery rate). The corrected *p* value was the *q* value. At the same time, the differential expression multiple was calculated based on the FPKM value, which was fold change (FC). The screening conditions were *p* ≤ 0.05 and FC ≥ 1.5 or FC ≤ 0.67.

### 2.9. Reverse Transcription-Quantitative Polymerase Chain Reaction (RT-qPCR)

Total RNA was extracted from liver tissue using an RNA simple total RNA kit and reversed to cDNA using the ReverTra Ace qPCR RT kit. RT-qPCR was performed on the ABI 7500 RT-PCR system (Applied Biosystems, Foster City, CA) under the following conditions: 95°C for 5 s, 60°C for 34 s (40 cycles), 95°C for 15 s, 60°C for 60 s, and 95°C for 15 s. The primer sequences are shown in Table 1. Each sample was performed 3 times. The relative expression level of genes was calculated using glyceraldehyde 3-phosphate dehydrogenase (GAPDH) as the internal control.

### 2.10. Western Blot

The liver tissues were homogenized with RIPA buffer. After centrifugation for 30 minutes at 12000 rpm, the homogenates were quantified using a BCA protein quantification kit. 30–50 micrograms of protein sample were separated by sodium dodecyl sulfate-polyacrylamide gel electrophoresis (SDS-PAGE) and transferred to a nitrocellulose (NC) membrane. The membrane was blocked with 5% bovine serum albumin in PBS and probed overnight at 4°C with the antibodies against CX3CL1 and GAPDH. After incubating with fluorescence anti-rabbit or anti-mouse antibody for 1 h at room temperature, the protein bands were scanned by the Odyssey infrared imaging system (LI-COR Biosciences, USA), and the densitometry analysis was calculated relative to GAPDH band using an Image J software.

### 2.11. Immunofluorescent Staining

The liver tissues were put into a Tissue-Tek OCT  embedding medium and snap-frozen in liquid nitrogen, and then, 10 *μ*m thick slices were stained with anti-CCL2 and anti-F4/80 immunohistochemical antibodies, following that fluorescent second antibodies like goat anti-rabbit IgG H&L (FITC) and donkey anti-rat IgG H&L (Alexa Fluor 647) were incubated. Next, the cell nuclei were stained with DAPI. Finally, the images were taken by a laser confocal microscope [18].

### 2.12. Flow Cytometry

To isolate liver macrophages from different groups, mice were anesthetized and perfused with SC-1 solution from the hepatic portal vein, then perfused with SC-2 solution containing pronase E (1 mg/30 ml) and SC-2 solution containing collagenase IV (1 mg/15 ml) successively. The liver was taken out, the liver capsule was torn gently, and then, the liver was kept in a culture dish on ice with 50 ml SC-2 solution containing 12.5 mg pronase E, collagenase IV, and 1 ml DNase I solution (adjust 2 mg/ml DNase I solution using GBSS/B); finally, the liver was stirred gently at 37°C for 20 min. Disaggregated cells were removed and pressed through 70 *μ*m cell strainers to obtain single cell suspensions, and hepatocytes were eliminated by centrifugation three times at 500 rpm for 3 min. Macrophages was purified by using successive gradient centrifugations on 34.5% and 14.5% Histodenz solution. Purified macrophage suspension was counted by an automatic cell counter and incubated immediately with CD45, CD11b, F4/80, Ly6C, CD86, and CD206 monoclonal antibodies for 30 min in the dark at 4°C. Also, gating strategies for leukocyte of different subclasses were proinflammatory Ly6C^high^ macrophages (CD45^+^F4/80^−^CD11b^+^Ly6C^high^ cells), restorative Ly6C^low^ macrophages (CD45^+^F4/80^−^CD11b^+^Ly6C^low^ cells), proinflammatory KC cells (CD45^+^F4/80^+^CD11b^−^CD86^+^ cells), and anti-inflammatory KC cells (CD45^+^F4/80^+^CD11b^−^CD206^+^ cells). At last, the stained cells were detected on the DxFLEX flow cytometer, and data were analyzed by Flowjo software.

### 2.13. Statistical Analyses

All experimental data processing was carried out using SPSS18.0 and the GraphPad Prism 8.01 software. The results were shown as the mean ± standard deviation (SD). The significance of differences between two groups was determined by the *t* test and one-way ANOVA. Significance was accepted at a *p* value of ≤0.05.

## 3. Results

### 3.1. FZHY Inhibited Liver Inflammation and Fibrosis in CCl4-Induced Liver Fibrosis Mice

**Figure 1:**
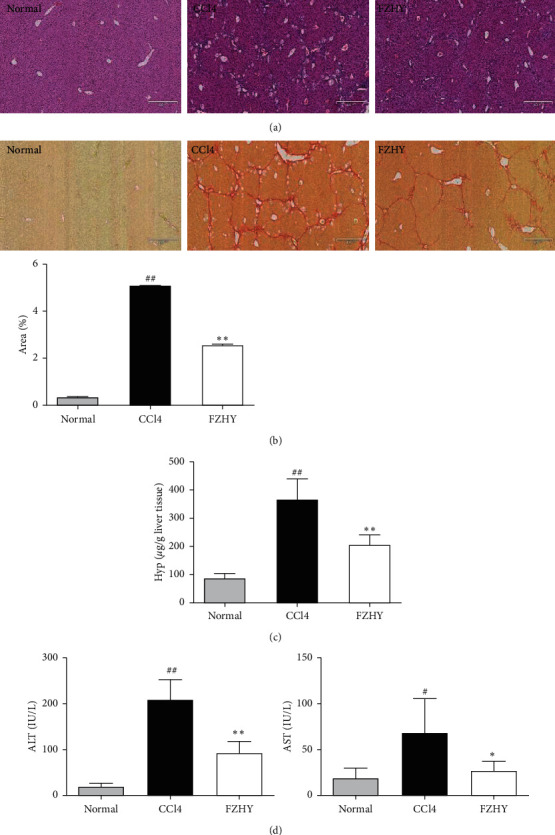
The prevention and curing effects of FZHY on CCl4-induced liver inflammation and fibrosis. (a) HE staining. (b) Sirius red staining. (c) Liver Hyp levels. (d) Serum ALT and AST levels. Results are expressed as means ± SD (*n* = 8). ^#^*p* < 0.05 and ^##^*p* < 0.01 vs. normal group, ^*∗*^*p* < 0.05 and ^*∗∗*^*p* < 0.01 vs. CCl4 group.

After mice were treated with carbon tetrachloride (CCl4) for 6 weeks, it was discovered that a large number of inflammatory cells were recruited in the portal area, numerous hepatocytes were degenerated and necrotized, and the liver lobule structure was destroyed in the liver tissues. Compared with the CCl4-induced liver fibrosis mice (CCl4 group), the infiltration of inflammatory cells and the necrosis of hepatocytes in the liver of Fuzheng Huayu recipe- (FZHY-) administered mice (FZHY group) were significantly reduced (Figure 1(a)). Collagen in the portal area was deposited extensively and extended to form fibrous septum in the liver tissues of CCl4 group mice, but the collagen deposition was significantly reduced and the fibrous septum became thinner or even disappeared in FZHY group mice (Figure 1(b)). Hydroxyproline (Hyp), which is a level major component of collagen proteins, was detected to assess liver fibrosis degree. Serum ALT and AST levels were measured to assess liver injury. The results showed that the liver Hyp levels (Figure 1(c)), serum ALT, and AST levels (Figure 1(d)) were significantly increased by CCl4 and significantly reduced by FZHY. These results showed that FZHY could markedly alleviate liver injury, inflammation, and fibrosis.

### 3.2. FZHY Exerted Its Anti-Inflammation Effect through TNF Signaling Pathway

In order to explore the cell signaling pathway and gene target which plays a key role in regulating macrophage phenotype, depending on which Fuzheng Huayu recipe (FZHY) exerted its anti-inflammation and antifibrosis effect, we carried out RNA-sequencing and transcriptome analysis.

**Figure 2:**
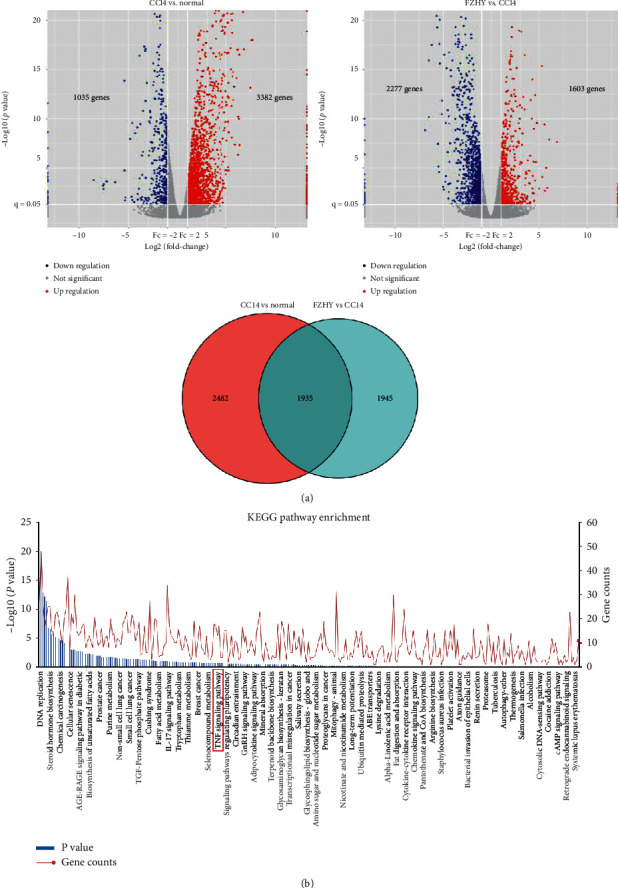
The analysis results of RNA-sequencing data (*n* = 3). (a) Volcano plot and Venn diagram of differently expressed genes. Red plots in volcano plot represent upregulated genes; blue plots in volcano plot represent downregulated genes. (b) KEGG pathway enrichment.

Sifting with *p* value ≤0.05, FC ≥ 1.5, or FC ≤ 0.67, 4,417 differential expression genes were obtained from 18,652 genes in CCl4-treated mice compared with control mice, in which 3,382 genes were upregulated significantly and 1,035 genes were downregulated significantly. 3,880 differential expression genes were obtained from 18,853 genes in FZHY-administered mice compared with CCl4-treated mice, in which 1,603 genes were upregulated significantly and 2,277 genes were downregulated significantly (Figure 2(a)). To elucidate the improvement mechanism of FZHY on CCl4-induced liver inflammation and fibrosis, further differential genes screening had been carried out. The differential expression gene set from the CCl4 group vs. normal group was intersected with differential expression genes set from the FZHY group vs. CCl4 group. We got an intersection set with 1,935 genes (Figure 2(a)). These genes should be involved in the effect of FZHY on alleviating liver damage and fibrosis.

To explore the key signaling pathways used by FZHY to inhibit liver inflammation and fibrosis in CCl4-induced hepatic fibrosis mice, we further input those 1,935 differentially expressed genes into the Kyoto Encyclopedia of Gene and Genomic (KEGG) database for pathway enrichment (http://bioconductor.org/biocLite.R). Enrichment results indicated that 310 signaling pathways were related to the inhibitory effects of FZHY on CCl4-induced mice hepatic fibrosis. Among them, TNF signaling pathway (*p*=0.213) was the most relevant pharmacological pathway for the regulation of macrophage recruitment and phenotype polarization. 13 differently expressed genes, such as CCL2, CXCL1, SELE, SOCS3, MLKL, LIF, RPS6KA5, TRAF3, TNFAIP3, ICAM1, MAP3K14, CSF1, and RPS6KA4, were distributed in the TNF signaling pathway (Figure 2(b)). These results showed that FZHY may suppress liver injury and inflammation through the TNF signaling pathway.

### 3.3. FZHY Affected the Expression of CCL2 and CX3CL1 in the Liver Tissues of CCl4-Induced Hepatic Fibrosis Mice

**Figure 3:**
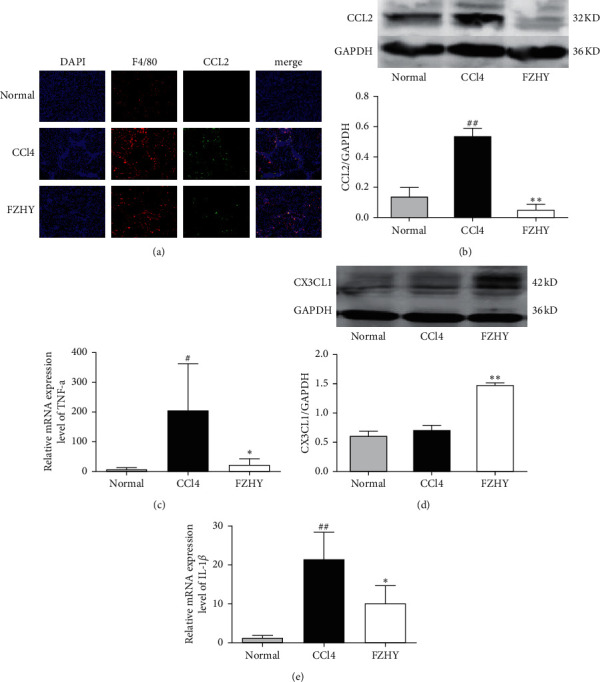
FZHY could regulate liver inflammatory cytokines and chemokines in liver tissue during liver fibrosis. (a) Immunofluorescence, (b, d) Western blot, and (c, e) qRT-PCR. Results are expressed as mean ± SD (*n* = 3). ^#^*p* < 0.05 and ^##^*p* < 0.01 vs. normal group, ^*∗*^*p* < 0.05 and ^*∗∗*^*p* < 0.01 vs. CCl4 group.

Chemokine factors, CCL2 and CX3CL1, play a key role on the recruitment and polarization regulation of intrahepatic macrophages [13]. Therefore, we further evaluated the expression of CCL2 and CX3CL1 genes which were members of the TNF signaling pathway in liver tissues. Immunofluorescence and western blot results showed that the expression of CCL2 was significantly increased in the liver tissues of CCl4-induced liver fibrosis mice and the distribution of CCL2 was mainly along the fibrous interval. The positive staining of CCL2 was obviously decreased in FZHY-administered mice (Figures 3(a) and 3(b)). Western blot results revealed that the expression of CX3CL1 was significantly increased by FZHY (Figure 3(d)). Meanwhile, the qRT-PCR results showed that the gene expressions of inflammatory factors IL-1*β* and TNF-*α* were increased by CCl4 and decreased by FZHY (Figures 3(c) and 3(e)). These results showed that FZHY could alleviate liver inflammation by decreasing CCL2 production and increasing CX3CL1 production in liver tissues.

### 3.4. FZHY Promoted CX3CL1 Expression and Suppressed CCL2 Expression in Primary Intrahepatic Macrophages of CCL4-Induced Liver Fibrosis Mice

**Figure 4:**
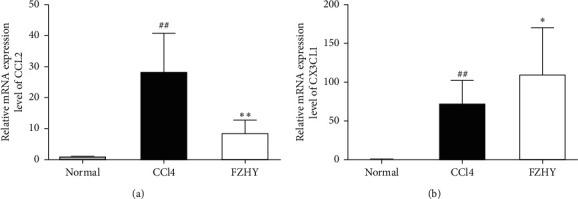
FZHY regulated the gene expression of chemokines CCL2 and CX3CL1 in primary intrahepatocyte macrophages. Results are expressed as mean ± SD (*n* = 3). ^##^*p* < 0.01 vs. normal group, ^*∗*^*p* < 0.05 and ^*∗∗*^*p* < 0.01 vs. CCl4 group.

The data showed above were from the detection of liver tissues; in order to elucidate whether Fuzheng Huayu recipe (FZHY) reduces liver inflammation and fibrosis through regulating macrophage phenotype polarization, we isolated primary intrahepatic macrophages and examined the gene expression of chemokines CCL2 and CX3CL1. As shown in Figure 4, the gene expressions of CCL2 and CX3CL1 in primary intrahepatic macrophages from CCl4-induced liver fibrosis mice were all significantly increased, while the gene expression of CCL2 in primary intrahepatic macrophages from FZHY-administered mice was markedly decreased, but the gene expression of CX3CL1 was further obviously increased. These results showed that FZHY could regulate macrophage polarization and chemotaxis through affecting chemokine secretion.

### 3.5. FZHY Significantly Reduced the Recruitment of Ly6C^high^ Macrophages and Upregulated the Ratio of Ly6C^low^ Macrophages by Suppressing the Proinflammatory Polarization of Kupffer Macrophages

In order to further study whether FZHY could inhibit the recruitment of bone marrow-derived proinflammatory macrophages through reducing CCL2 expression and promote the polarization of restorative macrophages through increasing CX3CL1 expression, we isolated primary intrahepatic macrophages for flow cytometry detection.

**Figure 5:**
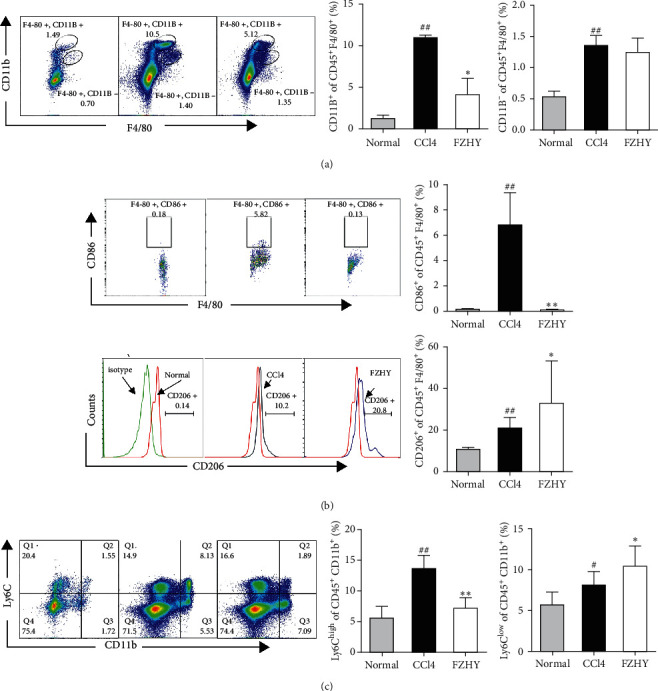
FZHY changed the macrophage phenotypes in the liver of CCl4-induced hepatic fibrosis mice and FZHY-administered mice. (a) Effect of FZHY on the ratio change of Kupffer and bone marrow-derived macrophages. (b) Effect of FZHY on the phenotype change of Kupffer macrophages. (c) Effect of FZHY on the phenotype change of bone marrow-derived macrophages. Results are expressed as mean ± SD (*n* = 4). ^#^*p* < 0.05 and ^##^*p* < 0.01 vs. normal group, ^*∗*^*p* < 0.05 and ^*∗∗*^*p* < 0.01 vs. CCl4 group.

The results showed that the ratio of peripheral mononuclear-derived macrophages (CD45^+^F4/80^+^CD11B^+^) was significantly increased by CCl4 and significantly decreased by FZHY. The ratio of liver resident macrophages-Kupffer cells (CD45^+^F4/80^+^CD11B^−^) was significantly increased by CCl4, and there was no significant change after FZHY was administered (Figure 5(a)). Further analysis on the phenotype of peripheral mononuclear-derived macrophages showed that the ratio of proinflammatory Ly6C^high^ macrophages (CD45^+^F4/80^+^CD11B^+^Ly6C^high^) in peripheral mononuclear-derived macrophages was significantly increased by CCl4 and significantly decreased by FZHY, and the ratio of restorative Ly6C^low^ macrophages (CD45^+^F4/80^+^CD11B^+^Ly6C^low^) was significantly increased by CCl4 and further significantly increased by FZHY (Figure 5(c)). Further analysis on the phenotype of Kupffer cells showed that the ratio of proinflammatory macrophages (CD45^+^F4/80^+^CD11b^−^CD86^+^) was significantly increased by CCl4 and significantly decreased by FZHY, and the ratio of anti-inflammatory macrophages (CD45^+^F4/80^+^CD11b^−^CD206^+^) was significantly increased by FZHY (Figure 5(b)). These results showed that FZHY could markedly inhibit the recruitment of bone marrow-derived proinflammatory Ly6C^high^ macrophages and increase the recruitment of bone marrow-derived restorative Ly6C^low^ macrophages through reducing the proinflammatory polarization of Kupffer macrophages.

## 4. Discussion

**Figure 6:**
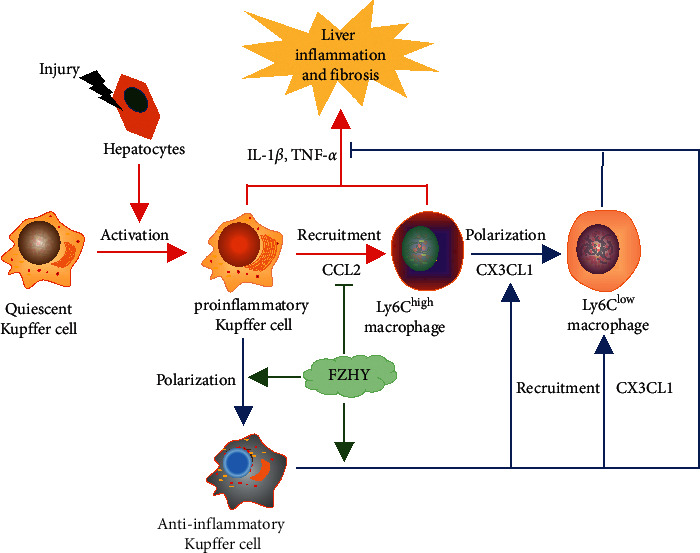
The prevention and treatment mechanism of FZHY on CCl4-induced hepatic fibrosis through regulating the recruitment and polarization of intrahepatic macrophages. FZHY reduced CCL2 gene expression of proinflammatory Kupffer cells which were activated in CCl4-damaged liver tissue, thereby reducing the recruitment of proinflammatory Ly6C^high^ macrophages. Meanwhile, FZHY promoted the phenotype polarization of proinflammatory Kupffer cells to anti-inflammatory Kupffer cells and increased CX3CL1 gene expression in intrahepatic macrophages, thereby it promoted the polarization and recruitment of restorative Ly6C^low^ macrophages. Anti-inflammatory Kupffer cells and restorative Ly6C^low^ macrophages exerts their anti-inflammation and antiliver fibrosis effects.

A large number of intrahepatic macrophages are located in the hepatic sinuses. It is estimated that there are 20–40 macrophages in every 100 hepatocytes in the liver of healthy animals [19]. Macrophages play a crucial role in the occurrence and progression of liver fibrosis [2]. Intrahepatic macrophages can be divided into two groups: Kupffer cells (KCs), which are residing in the liver, and peripheral mononuclear-derived macrophages, which are recruited from peripheral blood. Kupffer cells (KCs) are originated from yolk sac-derived specific progenitor cells and settled in the hepatic sinusoids. Normally, KCs are stationary and do not migrate [20]. They maintain the stability of the intrahepatic environment by identifying, phagocytizing, and degrading cell fragments, foreign bodies, and pathogens [21]. Stimulated by various extrahepatic pathogen associated molecular patterns (PAMPs) such as LPS and intrahepatic damage associated molecular patterns (DAMPs) from damaged hepatocytes and bile duct cells, KCs are polarized to proinflammatory CD86^+^-KCs. CD86^+^-KCs secrete enormous inflammatory cytokines, such as TNF-*α*, IL-1*β*, IL-6, and so on, which damage liver tissue and lead to liver inflammation and fibrosis. Meanwhile, the activated KCs recruited a large number of proinflammatory Ly6C^high^ macrophages from peripheral blood by releasing monocyte chemotactic protein 1 (CCL2). Proinflammatory Ly6C^high^ macrophages highly expressed CCR2, which combined with CCL2 expressed by KCs and infiltrated the liver [22]. Proinflammatory Ly6C^high^ macrophages secrete a large number of inflammatory factors, which further promote the damage of liver tissue [13]. Therefore, inhibiting the inflammatory polarization of KCs to reduce the recruitment of proinflammatory Ly6C^high^ macrophages is of great significance for the prevention and treatment of liver fibrosis (Figure 6).

Our results showed that Fuzheng Huayu recipe (FZHY) could significantly reduce the degree of CCl4-induced liver inflammation and improve the degree of CCl4-induced liver fibrosis in mice. At the same time, the data from RNA-seq of liver tissues showed that there were 1,935 differential expression genes among normal group mice, CCl4 group mice, and FZHY group mice. Enrichment analysis was carried out on these genes with KEGG database, and 310 pathways related to antiliver fibrosis effects of FZHY were discovered. TNF signaling pathway, which is the key pathway related to macrophage chemotaxis and inflammatory factor secretion, is one of them. These results indicated that FZHY might achieve its antifibrosis effects by regulating the recruitment and polarization of liver macrophages through the TNF signaling pathway. Among TNF signaling pathway-related genes, the expressions of CCL2, CXCL1, SELE, SOCS3, MLKL, LIF, RPS6KA5, TRAF3, TNFAIP3, ICAM1, MAP3K14, CSF1, and RPS6KA4 genes were significantly increased in CCl4 group mice and decreased in FZHY group mice and the expression of CX3CL1 gene was increased in CCl4 group mice and further increased in FZHY group mice.

CCL2, one of the differentially expressed genes in TNF signaling pathway, is a key chemokine for recruitment of peripheral inflammatory monocytes [23]. Proinflammatory Ly6C^high^ monocytes are recruited by CCL2, leading to further expansion of liver inflammation by releasing inflammatory factors and promoting the progression of liver fibrosis [24]. CCL2 is usually secreted by proinflammatory Kupffer macrophages in the liver. Our results discovered that the ratio of Kupffer macrophages (CD45^+^F4/80^+^CD11b^−^) in the liver of FZHY group mice was not obviously different from those of CCl4 group mice, but the ratio of proinflammatory Kupffer macrophages marked by CD86^+^ was significantly decreased and the ratio of anti-inflammatory Kupffer macrophages characterized by CD206^+^ was significantly increased. The data further showed that CCL2 protein production in liver tissues and CCL2 gene expression in primary intrahepatic macrophages were notably decreased by FZHY. Correspondingly, the proinflammatory Ly6C^high^ macrophages (F4/80^+^CD11b^+^Ly6C^high^) which were developed from Ly6C^high^ monocytes were also significantly reduced by FZHY. These results demonstrated that FZHY could inhibit the recruitment of proinflammatory Ly6C^high^ macrophages through repressing the secretion of CCL2 in proinflammatory Kupffer macrophages.

CX3CL1, which is also one of the differentially expressed genes in TNF signaling pathway, is in charge of the recruitment of restorative Ly6C^low^ monocytes which will develop to restorative Ly6C^low^ macrophages in the liver. And, CX3CL1 will also promote the polarization of proinflammatory Ly6C^high^ macrophages to restorative Ly6C^low^ macrophages. Restorative Ly6C^low^ macrophages are beneficial to the conversion of inflammation and liver fibrosis [13]. The results showed that CX3CL1 protein production in liver tissues and CX3CL1 gene expression in primary intrahepatic macrophages were significantly increased by FZHY. Correspondingly, the restorative Ly6C^low^ macrophages (F4/80^+^CD11b^+^Ly6C^low^) were also increased by FZHY. These results suggested that FZHY might promote the recruitment and polarization of restorative Ly6C^low^ macrophages by increasing the CX3CL1 gene expression in intrahepatic macrophages.

## 5. Conclusions

In conclusion, it is clear that Fuzheng Huayu recipe (FZHY) could regulate the polarization and recruitment of intrahepatic macrophage through decreasing CCL2 gene expression and increasing CX3CL1 gene expression, thereby showing its prevention and treatment effects on liver inflammation and fibrosis (Figure 6).

## Figures and Tables

**Table 1:** PCR primer sequence.

Gene	Forward primer	Reverse primer
*β*-actin	TGACGAGGCCCAGAGCAAGA	ATGGGCACAGTGTGGGTGAC
CCL2	ATTGGGATCATCTTGCTGGT	CCTGCTGTTCACAGTTGCC
CX3CL1	CTGCCCTCACTAAAAATGGTGG	AATGTGGCGGATTCAGGCTT
IL-1*β*	GCAACTGTTCCTGAACTCAACT	ATCTTTTGGGGTCCGTCAACT
TNF-*α*	CAGGCGGTGCCTATGTCTC	CGATCACCCCGAAGTTCAGTAG

## Data Availability

The dataset supporting the conclusions of this study is publicly available.
